# Three-Dimensional Printed Devices in Droplet Microfluidics

**DOI:** 10.3390/mi10110754

**Published:** 2019-11-04

**Authors:** Jia Ming Zhang, Qinglei Ji, Huiling Duan

**Affiliations:** 1 State Key Laboratory for Turbulence and Complex Systems, Department of Mechanics and Engineering Science, BIC-ESAT, College of Engineering, Peking University, Beijing 100871, China; 2 Department of Production Engineering, KTH Royal Institute of Technology, SE-100 44 Stockholm, Sweden; qinglei@kth.se; 3 Department of Machine Design, KTH Royal Institute of Technology, SE-100 44 Stockholm, Sweden; 4 CAPT, HEDPS and IFSA Collaborative Innovation Center of MoE, Peking University, Beijing 100871, China

**Keywords:** 3D printing, additive manufacturing, droplet, emulsion, microfluidics

## Abstract

Droplet microfluidics has become the most promising subcategory of microfluidics since it contributes numerous applications to diverse fields. However, fabrication of microfluidic devices for droplet formation, manipulation and applications is usually complicated and expensive. Three-dimensional printing (3DP) provides an exciting alternative to conventional techniques by simplifying the process and reducing the cost of fabrication. Complex and novel structures can be achieved via 3DP in a simple and rapid manner, enabling droplet microfluidics accessible to more extensive users. In this article, we review and discuss current development, opportunities and challenges of applications of 3DP to droplet microfluidics.

## 1. Introduction

Droplet microfluidics is one of the most important subcategories of microfluidics, which creates and manipulates discrete droplets through immiscible multiphase flows inside microchannels [1]. Droplet microfluidics mainly refers to chip fabrication, droplet formation, manipulation and applications [2]. Its rapid development contributes a significant number of applications to various fields [3]. Conventional chip fabrication methods are usually complicated and expensive, which hinders droplet microfluidics further development. Thus researchers are devoted to developing new fabrication methods bearing with simple and low-cost manner. Recent development of three-dimensional printing (3DP), or additive manufacturing (AM), technologies open a new door for fabrication of microfluidic devices. It was developed in early 1980s [4], which featured layer-by-layer fabrication process. 3DP is attractive for the fabrication of microfluidic devices based on the following reasons: (1) 3DP provides freedom of designing novel structures and capability of creation of truly three-dimensional (3D) structures. (2) It is easier to learn and operate whereas conventional methods usually require professional knowledge and additional training to understand. (3) Computer-aid as a center role integrating digital design, automated fabrication and fast prototyping is the next generation manufacturing mode, which is able to greatly reduce the production cycle life time and make it possible for cloud manufacturing.

Therefore, the application of 3DP techniques in microfluidics experienced explosive growth in recent years and many excellent reviews have reported 3DP development in microfluidic community [5,6,7,8,9,10,11]. To our best knowledge, no review works have dedicated exclusively to 3DP applications in droplet microfluidics which is the most important subcategory of microfluidics. Most excellent reviews summarize relevant studies based on different printing methods and discuss from manufacturing view. This review will focus on discussion of 3DP employment in droplet microfluidics from droplet dynamics perspective: (i) A brief summary of droplet microfluidics is first introduced, especially focusing on droplet generation methods including both passive and active methods. (ii) A brief summary of printing methods exclusively available for droplet microfluidic is then conducted. (iii) Studies on 3DP droplet microfluidics are categorized based on the manner of droplet formation and manipulation. (iv) The challenges and opportunities for 3DP development in droplet microfluidics are discussed.

## 2. Droplet Microfluidics

**Figure 1:**
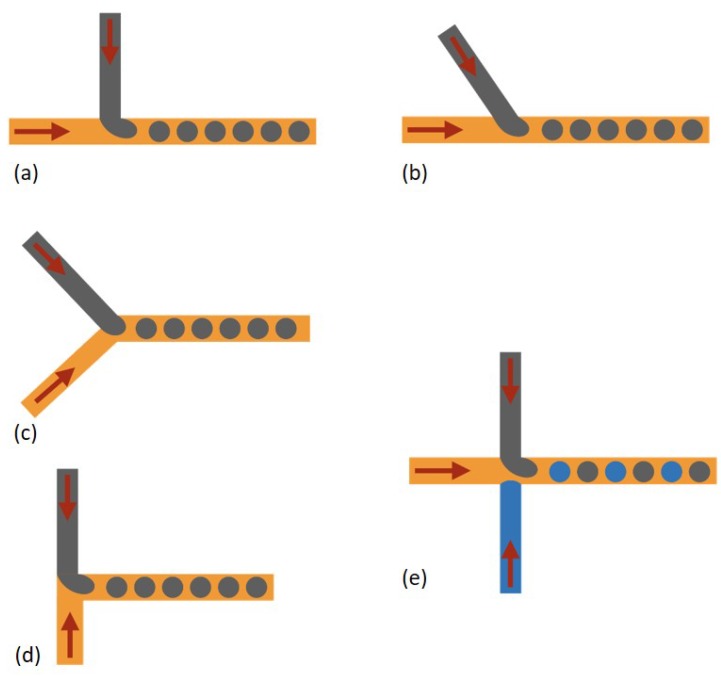
Cross-flow for droplet generation. (**a**) T-junction. (**b**) and (**c**) Y-junction. (**d**) Head-on junction. (**e**) Alternating T-junction.

**Figure 2:**
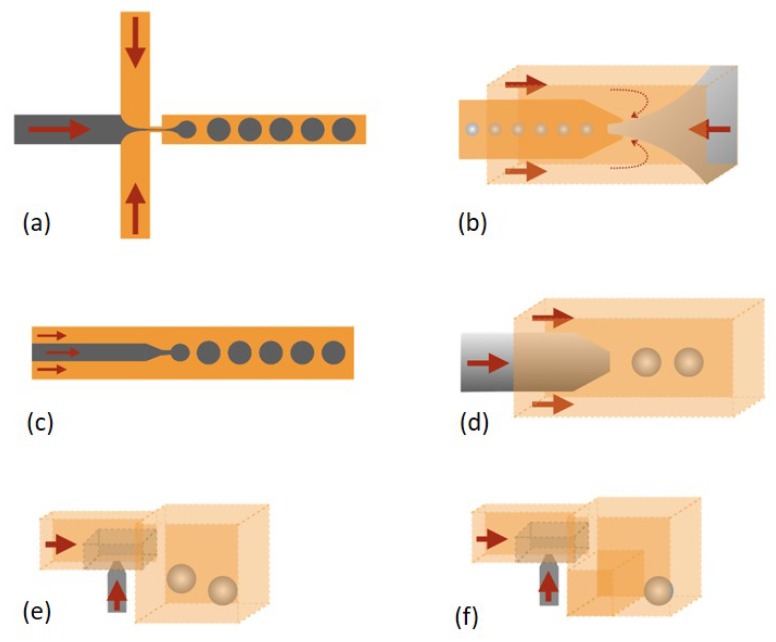
(**a**) Planar flow-focusing. (**b**) 3D axisymmetric flow-focusing. (**c**) Planar co-flow. (**d**) 3D axisymmetric co-flow. Step emulsification: (**e**) One step. (**f**) Two steps, or microchannel emulsification.

Various fabrication techniques for droplet microfluidic devices have been developed. Molding replication, micromachining and assembly of glass capillaries are three main methods for fabrication of droplet microfluidic devices [12]. Different structures for droplet formation were studied in the meantime. Co-flow, cross-flow and flow-focusing are three typical structures for droplet generation. T-junction (Figure 1a) is the mainly reported structure in cross-flow [13], as well as some variation structures originated from the T-junction such as Y-junction [14,15] (Figure 1b,c), head-on junction [16] (Figure 1d) and alternating T-junction [17,18] (Figure 1e) were reported. For co-flow and flow-focusing, two-dimensional (2D) [19] and 3D axisymmetric structures [20,21,22] were reported, respectively. An orifice in flow-focusing can facilitate the dispersed phase pinch-off (Figure 2a,b). The dispersed phase and continuous phase come from the same direction in co-flow (Figure 2c,d) [23,24,25]. In addition, step emulsification methods have also been reported (Figure 2e,f) [26,27,28,29,30,31]. Droplet generation mainly depends on the channel geometry and is less sensitive to flow rates and fluid properties. Therefore, the high monodispersity of droplets can be achieved, and also high-throughput droplet production can be achieved through parallel droplet generators. Besides the above methods, membrane methods have also been applied for producing droplet at industrial scales [32,33,34]. The dispersed phase goes through a porous membrane and then is pinched off by the continuous phase coming from perpendicular direction. In conclusion, the hydrodynamic pressure is the main force to passively produce droplets [35].

**Figure 3:**
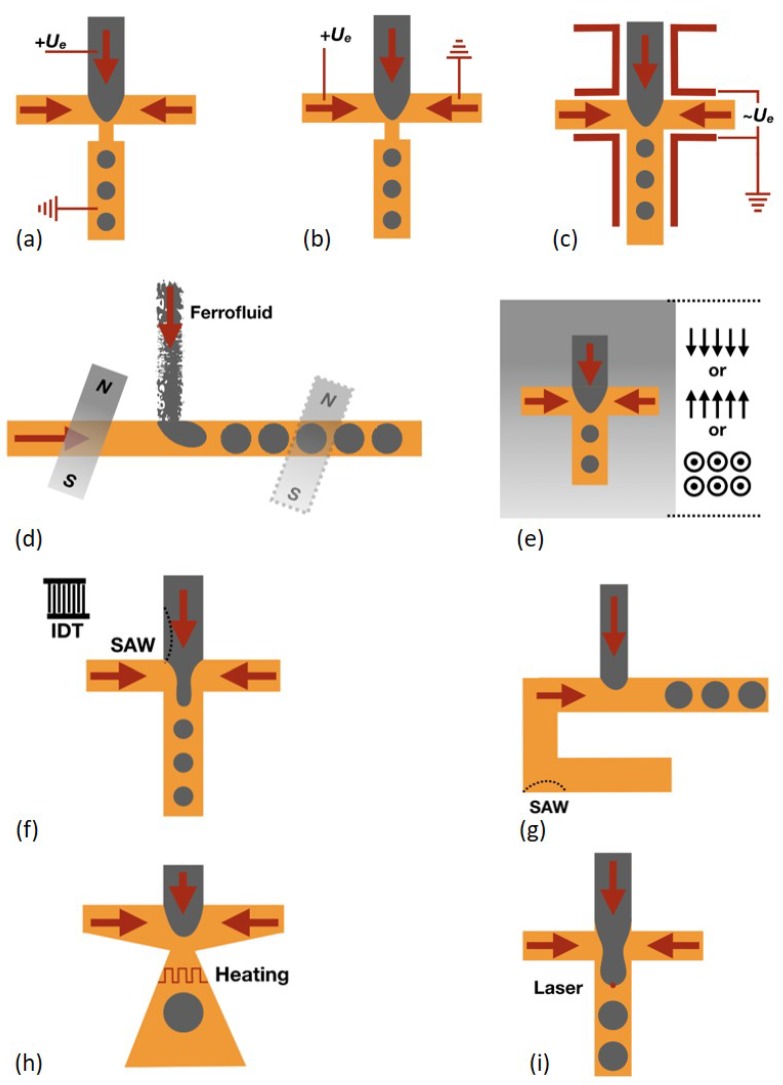
Electrical control methods: (**a**) Electrical field parallel to the flow. (**b**) Electrical field perpendicular to the flow. (**c**) Electrodes arrangement without contact with fluids. Magnetic control methods: (**d**) Magnet placed either upstream or downstream. (**e**) Magnet can be placed parallel and perpendicular to the flow direction or out-of-plane. Acoustic control methods: (**f**) SAW affects the liquid-liquid interface. (**g**) SAW affects the continuous phase. Thermal control methods: (**h**) Resistive heater at the junction. (**i**) Laser induced thermal effect on the droplet.

Besides passive methods, active external actuations including electrical, magnetic, thermal, acoustic and mechanical methods have also been used for droplet generation [36], which can provide more delicate control such as on-demand droplet generation and faster response time than passive methods. Here we only focus on the chip design and structures, which will be related to exploring the potentials of the 3DP technique. More details can be found in other excellent reviews. Electrical control methods were first developed, and both direct current (DC) [37] and alternating current (AC) [38] were used. The electrodes directly contact with the liquids [37] or through a metal wire contact with the liquids [39]. The electric field direction is parallel to the flow direction (Figure 3a) in most experimental cases and the one perpendicular to the flow direction was numerically studied [40] (Figure 3b). The additional electric stress on the water-oil interface promotes the dispersed phase pinch-off and decreases the droplet size. Tan et al. [38] proposed a method with which electrodes do not need to contact the liquids. Four electrodes are aligned at both upstream and downstream positions respectively (Figure 3c). A high frequency of alternating current facilitates the water droplet production.

Magnetic control methods have also been developed due to their non-contact control capability. A ferrofluid which contains nanomagnetic particles is always used in this method. It can be magnetized by external magnetic field and demagnetized when the external magnetic field is off. The external magnetic field can be applied by both magnet [41] and electromagnet [42], and various arrangement of magnetic fields was reported. Either upstream or downstream can the magnetic field be placed [43], and either perpendicular [44] or parallel [42,44] to the flow direction can the magnetic field also be placed (Figure 3d). Even the out-of-plane magnetic field can be applied [45] (Figure 3e). Droplet generation behavior is varied with different magnetic field arrangement. Another non-contact control method, i.e., acoustic control method, was also reported. The surface acoustic wave (SAW) generated by an interdigitated transducer (IDT) can affect the liquid pressure and liquid-liquid interface [46]. SAW can be applied to affect the droplet pinch-off behavior [47] (Figure 3f) or the pressure of the continuous phase [48] (Figure 3g) and then influence the droplet formation behavior. In addition, viscosity and interfacial tension of most liquids decrease with increasing temperature. Therefore, thermal control of droplet generation is possible. A resistive heater is located at the junction where droplets are produced to vary the fluid properties and then to alter the droplet generation [49] (Figure 3h). In addition, a focused laser beam can accurately locally heat the water-oil interface during the droplet creation to tune the droplet generation behavior [50] (Figure 3i).

**Figure 4:**
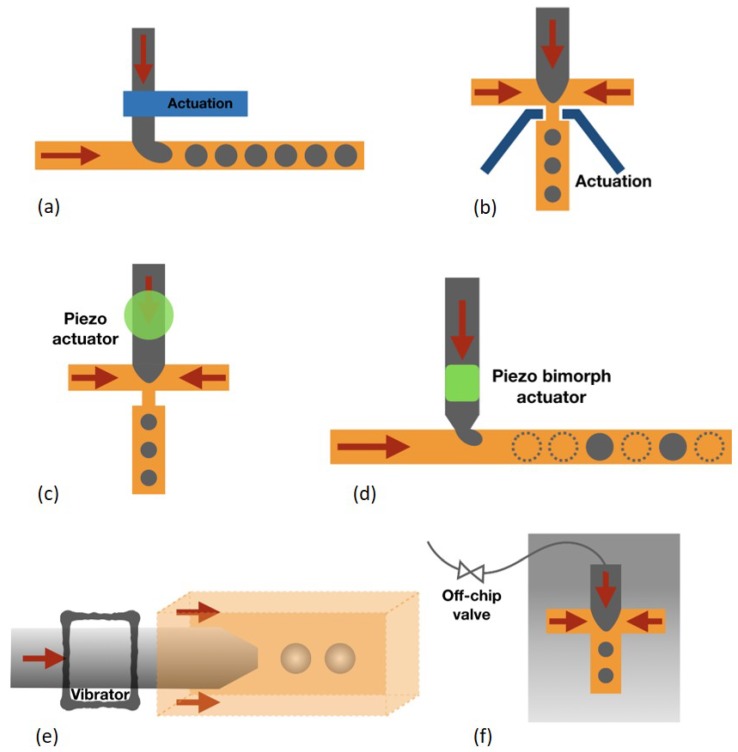
Pneumatic control methods: (**a**) One-side actuation channel. (**b**) Two-side actuation channels. Piezo actuation: (**c**) Piezoelectric disc under the dispersed phase. (**d**) Piezo bimorph actuator for on-demand droplet generation. Off-chip vibration: (**e**) A mechanical vibrator vibrates the microtubing. (**f**) Off-chip valves control dispersed phase flow.

Mechanical control methods refer to physical deformation of the channel resulting from pneumatic and piezoelectric actuation. The actuation channel is usually made of elastic materials such as PDMS which is easily to deform. The actuation channel through pneumatic control can be placed either upstream [51] (Figure 4a) or at the junction [52] (Figure 4b). Chen et al. [53] reported that a train of valves as choppers can cut the dispersed phase stream into multiple droplets. Furthermore, piezoelectric actuation is also used due to its fast response time. A piezo bimorph actuator was placed aside the dispersed phase [54] (Figure 4c). The actuation introduced by this actuator can inject droplets into the continuous phase, which enables droplet generation on-demand. A piezoelectric disc can also be mounted on the microfluidic device [55] (Figure 4d). The fast vibration introduced by this disc can result in an earlier breakup of the water-oil interface and thus smaller droplets can be produced. In addition, the vibration can also be introduced by off-chip methods. Zhu et al. [56,57] (Figure 4e) reported that a mechanical vibrator can hold the flexible microtubing to perturb the dispersed phase, which enables jets breaking into droplets. Mechanical valves can also be externally connected to microfluidic devices for on-demand droplet formation (Figure 4f). The droplet size depends on the valve on and off duration and this on and off cycle can be programmed to achieve on-demand droplet generation.

In addition, more complex droplet formation including multicomponent droplets, double-emulsion droplets and multiple-emulsion droplets was also reported [58]. In general, the design and fabrication of droplet microfluidic chip experienced development from 2D simple structures to 3D complex structures. 3DP that in nature has the capability of creating complex and 3D structures can play an important role in droplet generation, which will be introduced in the following sections.

Droplets need to be further manipulated after generation [59], to achieve desirable functions including coalescence, sorting, splitting, mixing, trapping, etc., which are crucial for lab-on-a-chip applications. In general, both passive hydrodynamic methods and active actuation methods through external forces introduced by electrical, thermal, mechanical and acoustic fields were proposed [60]. 3DP that is able to fabricate more complex structures and integrate various functional materials to introduce external fields can open a door for new droplet manipulation strategies, which will be also discussed in the following sections.

## 3. Available 3D Printing Techniques for Droplet Microfluidics

**Figure 5:**
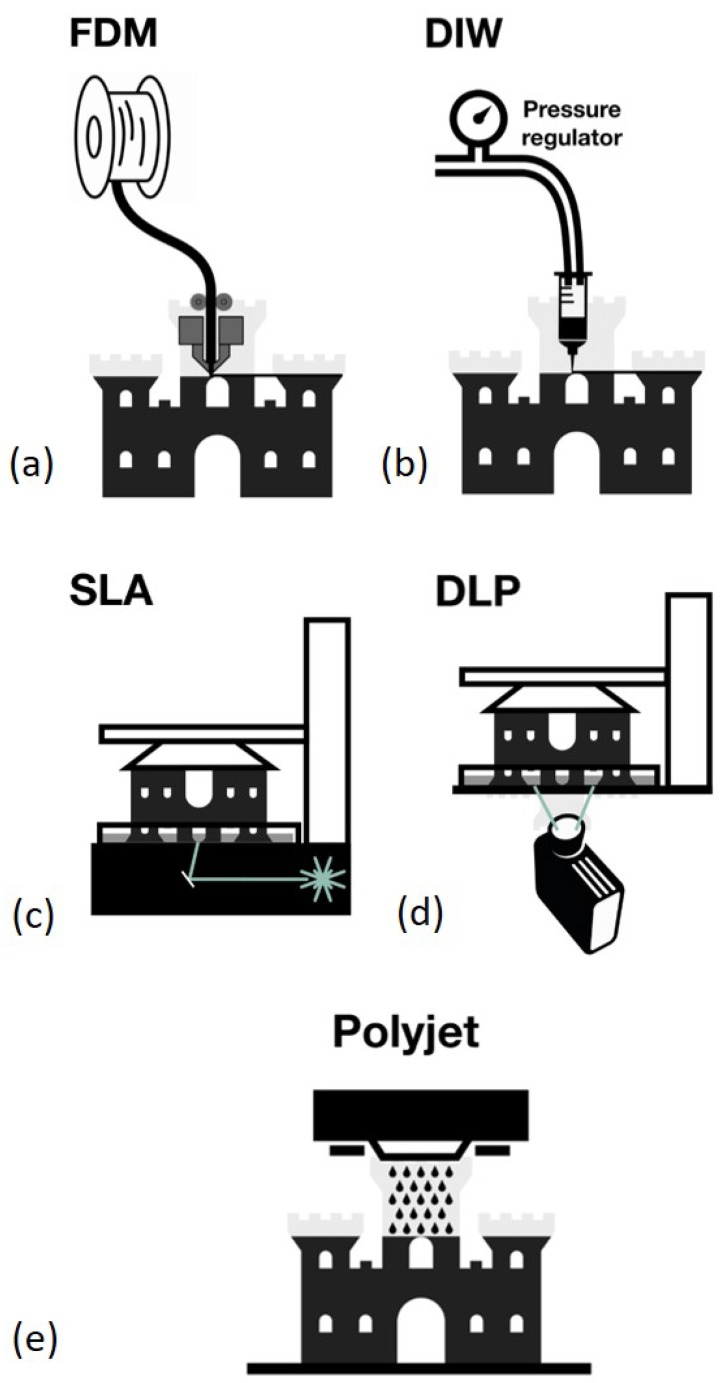
Current 3DP techniques applied in droplet microfluidics. (**a**) Fused deposition modeling (FDM). (**b**) Direct ink writing (DIW). (**c**) Stereolithography (SLA). (**d**) Digital light processing (DLP). (**e**) Photopolymer inkjet printing (Polyjet).

3DP can be classified based on the manner of the raw material deposition. The most widely one is the extrusion-based method, including fused deposition modeling (FDM) and direct ink writing (DIW) (Figure 5a,b) [61]. They were used for fabrication of microfluidic devices recently. A thermoplastic material is heated in the printing nozzle and then is extruded and cooled down immediately to form a structure layer for FDM. The printing nozzle can move in three dimensions and the object is built layer by layer. For DIW, the extrusion material is usually liquid which is driven by the pressure and then solidified by photocure or chemical crosslinking. The advantage of this method lies in its low-cost, simple operation and the wide range of materials available for printing, which shows its great potential in fabrication of various functional microfluidic devices. The disadvantage of this method is the relatively poor resolution and transparency compared with other 3DP techniques, which limit its applications in droplet microfluidics [62].

Another fast growing 3DP method is the vat photopolymerization, mainly including stereolithography (SLA) and digital light processing (DLP) (Figure 5c,d) [63]. Liquid photosensitive resin is usually used as the cured material and ultraviolet (UV) light is used as the light source. For SLA, a laser beam scans the liquid resin and its tracks determine the layer structure which is cured on the lift. Then the lift elevates for laser to scan another liquid layer which is then cured on the previous layer. Such steps are repeated until the whole object is built. For DIW, a laser or a UV lamp is used to cure the liquid photosensitive resin. The liquid resin is exposed to the light through a mask which determines the layer structure. One layer structure is directly formed by one time exposure instead of laser scanning. The object is then built layer by layer through multiple exposures. Digital mirror devices are used here to guide the light beam and form the pattern. The mask is not necessarily physical and is usually digital. It is similar to projecting images through an office projector. This method based on photocurable polymer resin usually offers better resolution and transparency, which are the most concerned parameters in droplet microfluidics. Therefore, this method is most widely used in droplet microfluidics [64].

It is noted that another method also based on photocurable polymer, inkjet 3DP or photopolymer inkjet printing (Polyjet) (Figure 5e), was also employed in microfluidics [65]. This 3D printer is similar to the commercial inkjet printer. The only difference is the inkjet material is photosensitive liquid resin in Polyjet. The liquid resin droplet is extruded through the inkjet nozzle and then is cured by the UV light to form a structure layer. The object is built layer by layer until finished. The distinct advantage of this method lies in its capability of multimaterial printing with a wide range of material properties such as hard and soft plastics and relatively high resolution, which is in nature suitable for fabrication of integrated microfluidic devices [66], whereas the high price of polyjet printers hinders its widespread applications in microfluidics.

The last major group of 3DP is the powder bed fusion-based methods [67], including selective laser sintering (SLS) and multijet fusion (MJF). Due to the relatively rough surface and non-transparency, these methods are rarely used in microfluidics and thus they are not the focus in this review. Following the short introduction of 3DP methods, 3DP studies based on droplet generation and manipulation perspective will be discussed.

## 4. 3DP Droplet Microfluidics

In this section, we will review the recent development of 3DP in droplet-microfluidics, which is first summarized in Table 1 and then details will be introduced in the following subsections.

### 4.1. Single Monolithic 3D-Printed Devices for Passive Droplet Generation

**Figure 6:**
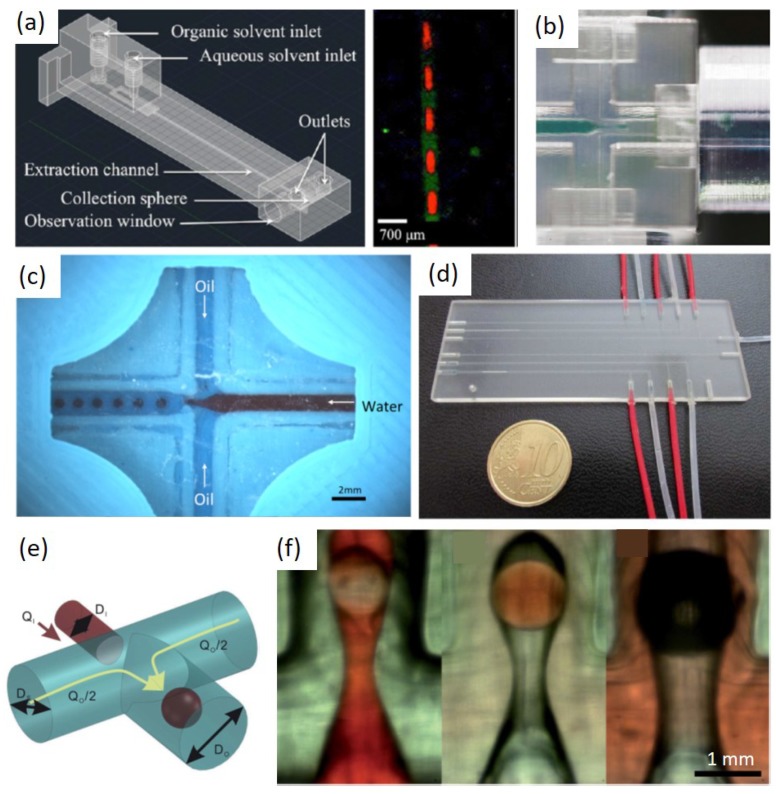
Current 3DP techniques applied in droplet microfluidics. (**a**) A flow-focusing structure printed by a desktop DLP printer. (**b**) A flow-focusing structure printed by SLA printing. (**c**) A flow-focusing structure printed by FDM printing. (**d**) A T-junction structure printed by Polyjet printing. (**e**) A schematic of non-planar structure printed by a desktop SLA printer and (**f**) different double emulsions were produced in this non-planar structure. Reproduce of [70,71,82,96].

First, the simple and straightforward idea is to directly use 3DP to fabricate structures for passive generation of droplets instead of conventional fabrication methods. Various printing methods were applied and typical droplet generation structures such as co-flow, flow-focusing and T-junction were 3D-printed. Shallan et al. [70] used a desktop printer to print a flow-focusing structure for droplet generation and perform liquid-liquid extraction (Figure 6a). Bhargava et al. [82] used a SLA printer to print both T-junction and flow-focusing structures for droplet generation (Figure 6b). Morgan et al. [96] used a FDM printer to print T-junction and flow-focusing structures for droplet creation (Figure 6c). Donvito used Polyjet printing to fabricate a T-junction droplet generator (Figure 6d). Romanov et al. [97] also used FDM printing to fabricate flow-focusing structures for droplet formation. Ohtani et al. [69] used SLA printing to print a co-flow structure for droplet generation. These works were pioneers of 3DP applications in droplet microfluidics and they more focused on the manufacturing advantage of 3DP compared with conventional methods. The structures used in their studies are mainly referred to conventional structures, which still formed 2D flow fields for droplet formation.

Later, 3D-printed structures which can introduce 3D flow fields for droplet generation were reported. Zhang et al. [71] reported a non-planar structure achieved by 3DP in which both W/O and O/W single emulsions and even different double emulsions (W/O/W and O/W/O) can be generated in a single device without need for special surface treatment (Figure 6e,f). This work exploited a remarkable and also nature advantage of 3DP in droplet microfluidics, which is able to create truly 3D structures in a simple manner for droplet generation. The inner phase was pinched off with no chance to contact the channel wall and therefore the additional surface treatment of channel wall can be avoided. The adaption of surface conditions to droplet generation mainly hinders droplet microfluidic applications in industry due to the lack of low-cost, simple and robust surface treatment methods. It requires complicated steps to create such 3D structures for conventional methods given that conventional methods are mostly 2D fabrication methods.

### 4.2. Hybrid and Modular 3D-Printed Devices for Passive Droplet Generation

Another idea that has been shown that 3D printed fluidic modules were used for arranging flow configurations [73], which were combined with tubing, glass capillaries and needles to form hybrid devices for droplet formation. Conventional methods such as glass capillary method require delicate manual process to assemble capillaries to form a suitable flow configuration for droplet generation, which is lack of being flexible and reproducible, whereas 3DP can be used to fabricate complex assembly modules. On the other side, due to the current resolution limitation, comparable channel dimensions with conventionally fabricated ones are difficult to achieve by 3DP, and therefore droplet sizes generated through 3D printed devices are usually larger. Such combination strategy with tubing or capillaries is able to not only alleviate the complexity of the manual assembly process but also produce smaller droplet sizes which are comparable to ones created by conventional methods.

**Figure 7:**
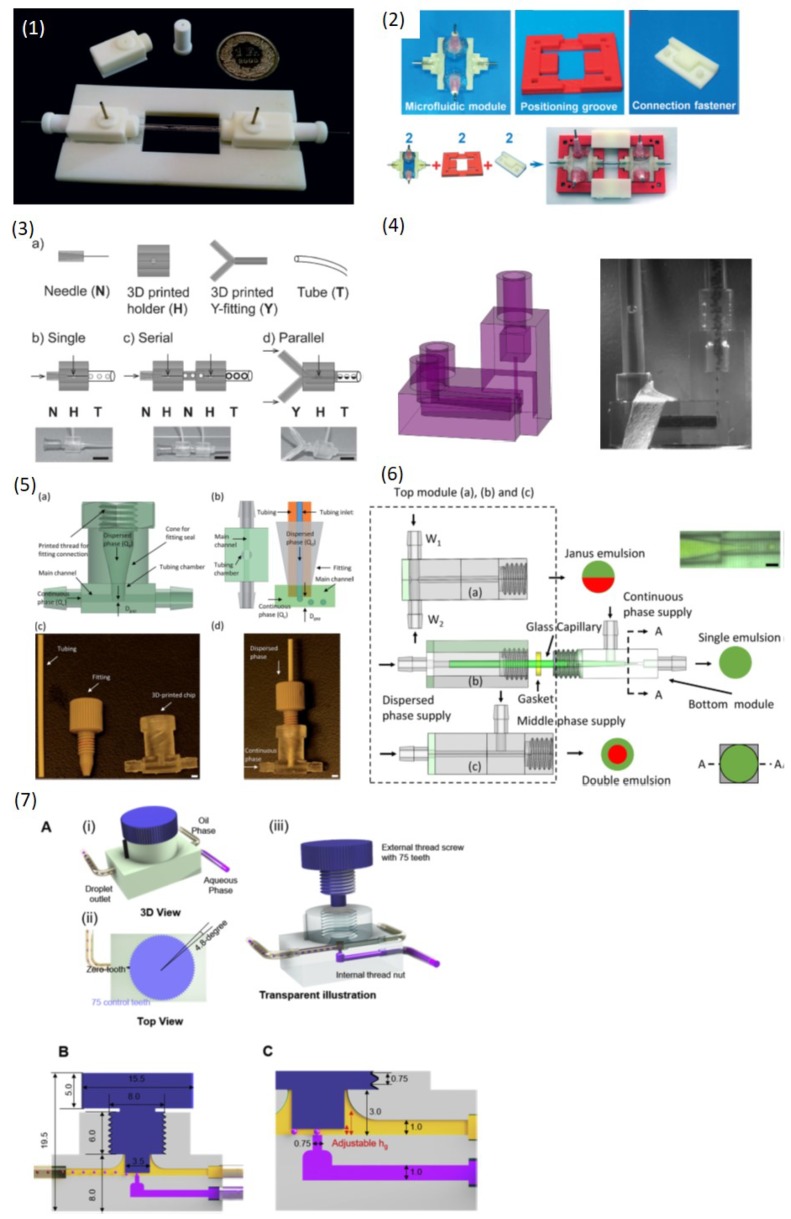
Hybrid droplet microfluidic devices. (**1**) A 3D-printed mold for flexible alignment of the glass capillary for generating double emulsions. (**2**) A 3D-printed groove and connection fastener for assembly of flow modules to produce multiple emulsions. (**3**) A 3D-printed holder and fitting for assembly of commercial needles and tubing for emulsion formation. (**4**) A 3D-printed chamber combined with commercial tubing to create smaller droplets. (**5**) 3D-printed various modules combined with a glass capillary to produce versatile emulsions. (**6**) A vertical T-junction structure consisting of a 3D-printed structure and commercial tubing. (**7**) A flexible T-junction structure formed by a 3D-printed chamber with a nut and a commercial screw. Reproduce of [72,75,78,79,80,81,93].

Martino et al. [79] printed a screw-nut structure to align the capillaries to produce double emulsions (Figure 7(1)). The position of the capillary nozzle can be flexibly varied for creation of different double emulsions. Meng et al. [80] printed positioning grooves and connection fasteners for flexible assembly of their flow-control modules (Figure 7(2)). Different flow-control modules can be easily assembled and disassembled for production of various higher-order multiple emulsions as well as for scale-up droplet production. Vijayan [75] printed various manifolds to connect and configure commercial low-cost needles and tubings for producing single and multiple emulsions (Figure 7(3)). Zhang et al. [93] used a desktop SLA printer to fabricate a tubing chamber, and tubing was inserted into this chamber to form a gap channel where droplet were produced (Figure 7(4)). The droplet size mainly depended on the tubing inner diameter (ID) and the gap channel, and thus down to 50 μm droplet can be generated through the relatively small tubing ID and gap channel, which overcame the difficulty of producing small droplets via 3D-printed device. Later Zhou et al. [81] used a DLP printer to fabricate different modules to assemble glass capillaries for emulsion creation (Figure 7(5)). The 3D-printed modules ensured the concentricity of capillaries with the channel and provided a plug-and-play assembly manner in which the glass capillaries can be easily changed. Thus a wide range of droplet sizes can be produced. The modules were also modular designed and single emulsions, double emulsions and Janus emulsions can be achieved by replacing different modules for introducing different liquid inputs. Riche et al. [72] vertically integrated the collection tubing with a horizontal flow module 3D-printed by a SLA printer to form a vertical T-junction structure (Figure 7(6)). When both dispersed and continuous phase entered the vertically oriented tubing, the dispersed phase segmented into droplets at the corner. The droplet size mainly depended on the collection tubing ID rather than the flow rates, which paved a way for scale-up droplet production. Nguyen et al. [78] proposed a flexible T-junction structure by combination of a commercial screw and 3D-printed fluid channels with a nut (Figure 7(7)). The droplets were produced at the gap which was formed by the screw and the channel wall. The gap distance can be simply tuned by rotating the screw and thus different droplet size can be obtained.

**Figure 8:**
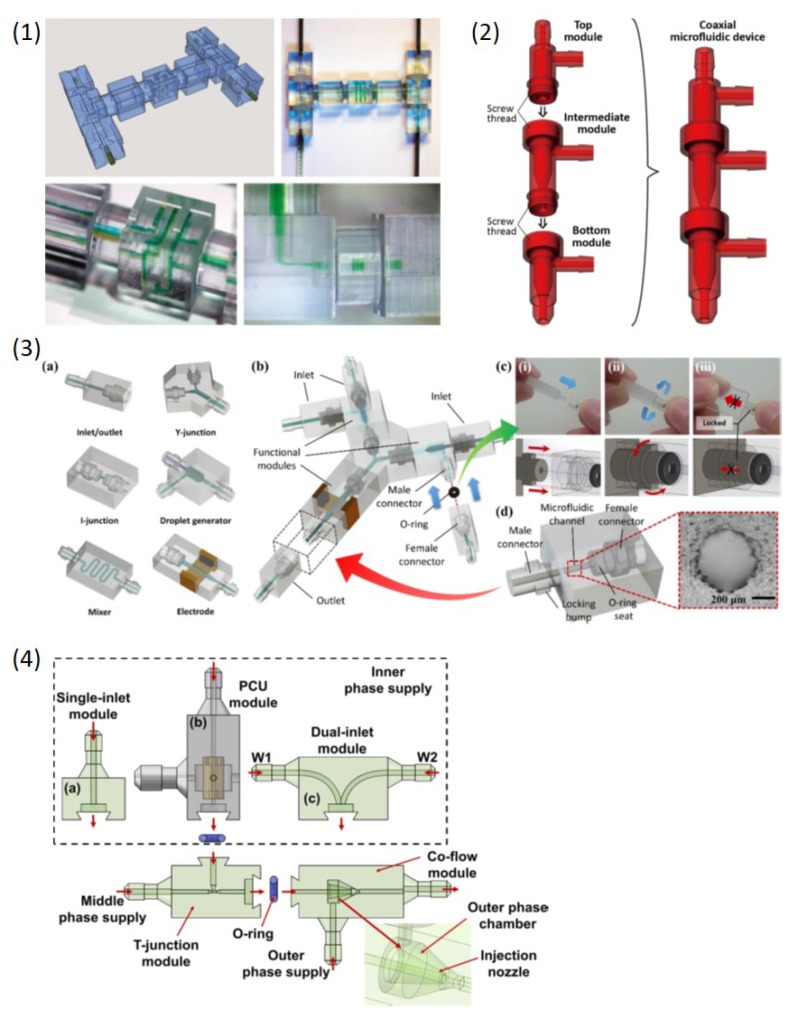
Modular droplet microfluidic devices. (**1**) 3D-printed modules were assembled to form a T-junction and a 3D helical mixer. Each module was connected with a male-male connector aligned with female-type ports. (**2**) Different 3D-printed modules were assembled to produce single and double emulsions. A notch structure with an O-ring connected each module. (**3**) Assembled 3D-printed modules to produce multiple emulsions with screw-thread as connection. (**4**) Various functional 3D-printed modules can be assembled to achieve different functions including droplet generation and manipulation. Male-female connectors with locking bump and O-rings connected each module. Reproduce of [77,82,92,94].

Furthermore, modular design and fabrication capability introduced by 3DP was used in droplet microfluidics, and different modular connection structures were introduced. Bhargava et al. [82] first used SLA printing to create modular standardized components and male-male connectors, which can be integrated to form various functional modules (Figure 8(1)). Both flow-focusing and T-junction modules can be flexibly assembled for droplet generation. Ji et al. [92] used a desktop SLA printer to print different building blocks including single-inlet modules and dual-inlet modules which can be flexibly integrated through easily detachable notch structures (Figure 8(2)). Versatile multiple emulsions can be produced through different modules. The monolithic coaxial nozzle was first successfully printed and both encapsulated number and components of droplets can be highly controlled. Later, Morimoto et al. [77] used SLA printing to print a coaxial nozzle as well. A top, intermediate and bottom module can be integrated through screw threads to produce double emulsions and multilayer fibers (Figure 8(3)). Song et al. [94] also applied the similar modular design to achieve different functions including generation of single and double emulsions (Figure 8(4)).

The hybrid and modular strategy summarized above liberates designers to build versatile droplet microfluidic systems and through such strategy 3DP advantage on droplet microfluidics was superbly demonstrated. Many novel methods and structures for droplet generation via 3DP which are not able or rather difficult to be achieved by conventional methods were reported. It greatly liberates designers for inventing novel structures and devices for droplet microfluidics.

### 4.3. Droplet Manipulation and Active Droplet Control

With the rapid development of droplet microfluidic applications, sophisticated and delicate control of the droplet generation and manipulation is highly desired. Therefore, active methods for control of droplet generation and manipulation were reported due to their better capability for controlling droplets compared with passive methods [35]. Electrical, thermal, magnetic, mechanical, pneumatic and acoustic control methods wereinvestigated in conventional droplet microfluidics and many excellent reviews have summarized related studies [36,59]. Recently some active components such as valves [85,88], pumps [86,87], and even classical Quake-style microvalves were successfully 3D printed [89], which demonstrated 3DP potentials in active control of droplets.

**Figure 9:**
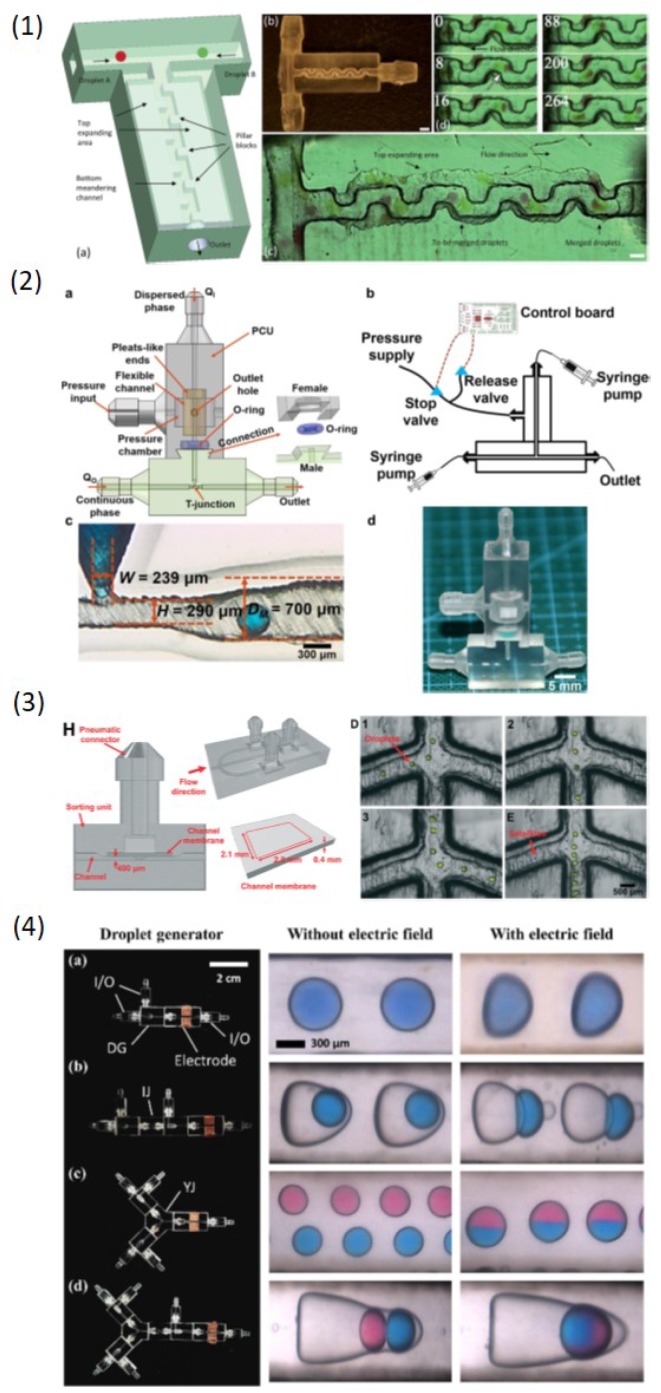
(**1**) Pneumatic control of droplet formation through a 3D-printed flexible membrane. (**2**) Pneumatic control of droplet sorting through a 3D-printed flexible valve. (**3**) Droplet coalescence in a 3D-printed channel consisting of a top layer for flow drainage and a bottom rail layer for droplet guide. (**4**) Droplet deformation, release and coalescence in a 3D-printed channel through an external electric field. Reproduce of [83,92,93,94].

However, only a handful of studies on using 3DP to produce and manipulate droplets in an active manner were reported. There is much room for development of 3DP in active control of droplets, especially for multimaterial 3DP which in nature have capability of integration of functional materials such as conductive materials and hard/soft materials. It is very convenient for multimaterial 3DP technology to introduce external fields to control droplets through these functional materials, whereas conventional methods require additional and complicated process to integrate such as electrodes, valves and other functional parts. Ji et al. [92] used multimaterial Polyjet printing to create an active droplet generator (Figure 9(1)). The generator monolithically consisted of a soft part (Tango Plus FLX930) and a surrounding rigid part (VeroClear). The soft part was pneumatically controlled and droplets were actively generated following with the excitation frequency. Furthermore, they demonstrated that double emulsions with different compositions or with different number of encapsulated droplets can also be generated using this active method.

On the other side, manipulation of droplets in 3D-printed devices including droplet coalescence and sorting has started to be reported. Zhang et al. [83] printed a monolithic sorting junction embedded with valves via multimaterial printing (Figure 9(2)). Through pneumatic control of valves to tune the flow resistance, droplet can be sorted into the desired channel. Zhang et al. [93] 3D-printed a 3D structure which consisted of two layers for highly efficient droplet coalescence (Figure 9(3)). The top layer drained flows for decelerating droplets to make them contact and bottom rail layer guided droplet motion to reduce the freedom of droplet motion to make sure the droplet contact. Thus droplet coalescence efficiency was enhanced in this way. Song et al. [94] bonded electrodes on the droplet generation module (Figure 9(4)). In the presence of an external DC electric field, single emulsions can be deformed and coalesced, and double emulsions can be ruptured to release the inner core droplets.

### 4.4. High-Throughput Droplet Generation

**Figure 10:**
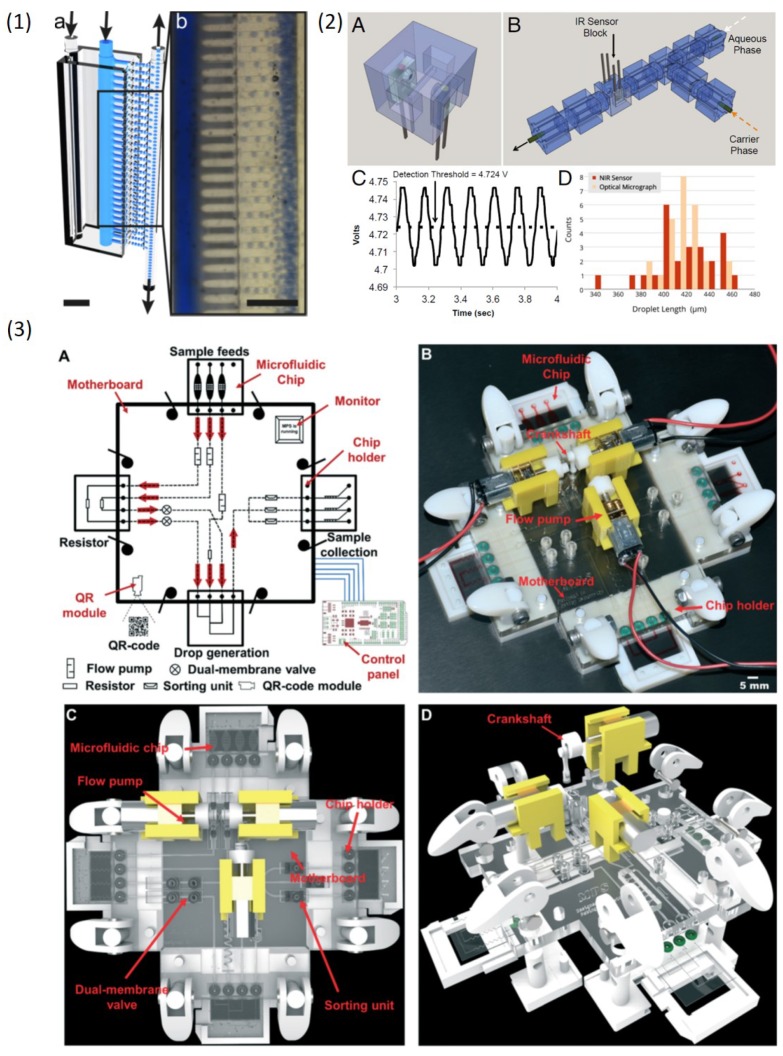
(**1**) 3D-printed parallel flow-focusing structures for high throughput production of emulsions. (**2**) A near-infrared sensor embedded on a 3D-printed device to monitor droplet generation. (**3**) An integrated milli/microfluidic processing system printed by Polyjet including droplet generation, detection, sorting and collection. Reproduce of [82,83,95].

Droplet microfluidic community always pursuit high yields of droplets since it is the prerequisite for droplet applications in industry. Therefore, many methods for high-throughput droplet generation were reported [98]. However, the structures for high-throughput droplet production are usually complicated and fabrication of such complicated structures via conventional 2D methods still remains challenging due to the inevitable multilayer fabrication process. The capability of 3DP for fabricating complicated structures has great potential in high-throughput droplet production. Femmer et al. [95] used the DLP printing method to create a 28 parallel flow-focusing structures (vertical stacking) to produce around 500 μm droplets and microgels at rates of 3 L/h (Figure 10(1)), which satisfies with industry requirements. Procedures such as alignment and bonding of multilayers in other conventional methods were completely eliminated.

### 4.5. Integrated System

3D-printed modules as building blocks to achieve modular design and fabrication of droplet microfluidic devices summarized in the previous section demonstrated 3DP capability of manufacturing complex modular systems. However, a highly integrated droplet microfluidic system fabricated by 3DP is still lacking. An integrated system that can process all droplet actions is highly desirable for biological and medical applications. Bhargava et al. [82] first showed that sensors can be integrated on the 3D-printed device (Figure 10(2)). A near-infrared emitter-receiver were mounted on a droplet generation module to monitor the droplet formation including droplet size and generation frequency. Cecil et al. [84] also integrated LED on a 3D-printed device for photometric detection. Recently, Zhang et al. [83] reported an integrated micro-millifluidic processing system which was manufactured by multimaterial 3DP (Figure 10(3)). Droplet generation, detection, sorting and collection can be achieved in their complex system, which shows 3DP great potentials in developing complicated and integrated system for droplet control.

### 4.6. Summary of Current 3DP Technique and Devices for Droplet Microfluidics

We introduced various studies on 3DP techniques applied in droplet microfluidics. Here, we briefly summarize current 3DP techniques only suitable for droplet microfluidics (Table 2), which is as a reference for ones might be interested in using 3DP to fabricate droplet microfluidic devices. Other 3DP techniques and materials not suitable for droplet microfluidics are not listed.

## 5. Challenge and Opportunities for 3DP in Droplet Microfluidics

In this section we will discuss the most concerned aspects for applying 3DP to droplet microfluidics. For example, the surface wettability and roughness of the channel is crucial for droplet microfluidics whereas they are less important for other microfluidic applications. We summarize the challenges of 3DP applications in droplet microfluidics in the following sections. The challenges are also the developing opportunities for 3DP in droplet microfluidics, and regarding improvement works for challenges are also summarized.

### 5.1. Resolution

The channel dimension is crucial for droplet microfluidics since the minimum droplet size mainly depends on the channel feature dimension. In general, the current 3DP technology cannot compete with conventional methods regarding the channel dimensions. Conventional methods can fabricate smaller channel and thus smaller droplets can be created. It is noted that the printing resolution provided by the 3D printer does not represent the minimum channel dimension which can be fabricated. For SLA printing, the uncured resin is rather difficult to remove since the capillary effects due to the small channel geometry. For FDM printing, the large size of the nozzle and filament make the printed channel large despite the high printing resolution of these printers. Therefore, the printable minimum channel dimensions are always larger than the printing resolution. Although current 3D-printed devices are still too large for many microfluidic applications, some great efforts were made recently to improve the capability of 3D printing smaller channels. Nordin’s group [99] customized a DLP 3D printer with a 385 nm LED and more efficient photoinitiators which can greatly improve the printable channel dimensions. The channel with cross-section 18 μm × 20 μm was successfully printed. Gale’s group [100] lowered the FDM printing nozzle close to the print surface and the extrusion lines were thus flattened and the line width was increased, which reduced the channel width. The actual channel width is around 100 μm smaller than the design width. In this way, down to 40 μm channels can be printed by FDM printing. Spence’s group [101] 3D printed enclosed microfluidic channels via Polyjet printing using liquid supports or membrane supports instead of photocurable supports, and the channel with cross-section 125 μm × 54 μm can be successfully fabricated. It was shown that 3D printed devices started to develop from millifluidic to truly microfluidic sub-100-μm [100,102].

### 5.2. Surface Wettability

The W/O droplet generation requires hydrophobic channel surface while O/W droplet generation requires hydrophilic channel surface. Thus, wettability of 3DP materials should be first considered for droplet microfluidics except truly 3D structures. The surface wettability of some printing materials used for fabrication of droplet microfluidic devices is listed in Table 3. The value of Stratasys Veroclear resin was measured by ourself. Effective coating materials and methods for surface treatment of the 3D-printed channel need to be further developed.

### 5.3. Transparency

Transparency of materials is also important for droplet microfluidic since the process of controlling droplet such as generation or manipulation usually requires optical observation. In general, vat photopolymerization provides better transparency while FDM printing offers poor transparency due to the scattering of light at the interface of the extrusion layers, even for the transparent FDM filaments. The device printed by SLA and DLP printing now can provide satisfactory transparency after the surface is polished. Recently, Folch’s group used 385 nm LED to cure the low-MW poly(ethylene glycol) diacrylate (MW 250) (PEG-DA-250) to print a highly transparent microfluidic device [108]. On the other side, some strategies for improving transparency of FDM printed devices were also reported. Bishop et al. [109] used a very thin side wall which reduced the number of printing layers to improve the channel transparency. Morgan et al. [96] embedded a glass window on the FDM printed device for optical observation, Romanov et al. [97] directly FDM printed structures on a glass slide and Bressan et al. [110] directly printed structures on a transparent PMMA slide. Recently, Gale’s group lowered the nozzle to flatten the extrusion lines which reduced the voids between lines and layers to improve the transparency of FDM printed device and 85% transparency can be achieved [100]. In short, the transparency issue of 3D-printed devices can be solved through different strategies.

### 5.4. Surface Roughness

Droplets will be ruptured or scratched during their transportation inside a too rough channel. Therefore, the surface roughness of the channel should be carefully considered. The surface roughness of 3D-printed device is varied depending on different 3DP fabrication methods. Generally, the channel surface printed by SLA and DLP printing is smoother than the one printed by FDM printing [111]. One possible post-processing step for FDM device is that printed devices can be put in organic solvent vapor environment for a while to erode small extrusions inside the channel. It is noted that on the contrary the surface roughness provided by FDM printing can be used for enhancing fluid mixing efficiency [112]. The surface quality printed by Polyjet printing depends on how clean one remove the supports. Too many support residuals on the channel surface will affect the droplet transportation inside. More detailed studies on the channel surface quality should be conducted. In summary, the current 3D-printed channel surface is acceptable for droplet motion although the surface quality is inferior to the one fabricated by conventional methods.

### 5.5. Biocompatibility

There are many biological and medical applications based on droplet microfluidics, and therefore material biocompatibility of droplet microfluidic devices is a primary concern. A significant number of studies on the conventional material biocompatibility were conducted, while only a small quantity of biocompatibility studies and reviews on 3DP materials were reported [113,114,115]. Generally, devices fabricated by extrusion-based 3DP have better biocompatibility than ones created by vat photopolymerization 3DP [116]. Some photoinitiators, photopolymers and additives used in vat photopolymerization 3DP have toxic effects. Commercial rein formulas are proprietary and thus in-depth biocompatibility studies on these resins are difficult to conduct. It is noted that Zhu et al. [117] and Macdonald et al. [118] showed that the leachate from polymerized parts (VisiJet Crystal polymer) has toxic effects on vertebrates and invertebrate model organisms, although this photopolymer was classified as a substance with favorable biocompatibility. The mechanism on toxic effects can be very complicated and more careful caution is required when 3D-printed devices are used for biological applications. Developing well-known biocompatible material to be 3D printable is an excellent way for improving 3DP biocompatibility. Folch’s group developed SLA printing with PEG-DA (MW250) [108] and Poly(dimethylsiloxane) (PDMS) [119] which are biocompatible materials. Biocompatible hydrogels for SLA printing [120,121] and biocompatible polymers for FDM printing [122] were also developed recently. In conclusion, novel biocompatible printing materials are highly desired and more thorough biocompatibility investigation of current available printing materials is also highly desired.

## 6. Conclusions

3DP droplet microfluidics is still at an early stage. SLA, DLP and FDM printing are three main printing methods applied in droplet microfluidics and each has their individual advantages and disadvantages. Currently, most studies focus on the droplet generation. 3DP offers greater freedom for the design and fabrication of droplet formation devices. The fabrication development experienced the single monolithic device, hybrid/modular device as well as the integrated system, and the droplet generation structure evolved to be truly 3D where surface treatment of channel wall can be avoided for different emulsion production. The droplet generation method developed from passive to active and high throughput manner. 3DP can play more important role in active and high throughput droplet generation by integrating various functional materials and introducing novel structures to the microfluidic device. External actuation fields can be introduced through functional materials integrated on the device, which is easily achieved by multimaterial 3DP. High throughput droplet generation always requires more complicated structures which are rather difficult to fabricate for conventional methods, whereas 3DP has the capability of creating such complex structures without intense labor. Furthermore, 3DP opens a door for new droplet manipulation strategies. Various novel 3D structures can be used for passively manipulating droplets, and available functional materials printed by multimaterial 3DP also liberate researchers to develop new active manipulation methods. These relevant studies were rarely reported and there is still much room for 3DP development in the above mentioned areas.

Printing resolution, printed channel quality and material biocompatibility still remain challenging, and many researchers are devoted to solving these limitations. These limitations will be no doubt improved and finally solved in the near future. By that time, 3DP advantage will be more prominent. 3DP represents the next generation manufacturing technology. Digital 3D design and rapid prototyping will completely change the current manufacturing manner. Design files can be easily shared with other distant collaborators and manufacturing process can also be supervised online. On the other hand, long cycle times of prototyping and fabrication can be greatly reduced. From design to fabrication of one microfluidic chip typically take days for conventional methods, while 3DP can finish the whole process in a couple of hours. Researchers will spend much less time verifying their ideas and the manufacturer will significantly reduce the production life cycle. Therefore, we envision that 3DP applications in droplet microfluidics or more general microfluidic fields will have a bright future.

## Figures and Tables

**Table 1:** A brief summary of recent 3DP development in droplet-microfluidics.

3DP Development	Specification	Reference
Monolithic	Single chips were produced and existing structures have recurred. Non-planar structures have also been reported.	[68,69,70,71]
Hybrid and modular	Combine tubing or glass capillaries to produce droplets. Smaller droplets can be produced. Flexible and modular assembly methods were reported. The advantage of 3DP in droplet-microfluidics was demonstrated.	[72,73,74,75,76,77,78,79,80,81]
Integrated system	Complicated and integrated devices were reported, whereas they are difficult to fabricate through conventional methods. The potentials of 3DP can be envisioned.	[82,83,84]
Droplet Manipulation and active control	Besides simple droplet generation, droplet manipulation and active control were reported.	[85,86,87,88,89,90,91,92,93,94]
High-throughput droplet production	3DP enables fast-prototyping parallel droplet generator which requires complicated fabrication process for conventional methods.	[95]

**Table 2:** Summary of current 3DP techniques suitable for droplet microfluidics.

	FDM	SLA or DLP	Polyjet
Principle	Extrusion	Photocuring	Inkjet
Material	Thermoplastic	Liquid photosensitive resin	Liquid photosensitive resin
Feature resolution	∼100 μm	∼25 μm	∼14 μm
Reported drop size	⪆100 μm	⪆50 μm	⪆100 μm
Advantage	Simple, low-cost and biocompatible	Transparent, smooth surface and high accuracy	Multimaterial, large printable dimension and high accuracy
Disadvantage	less transparent, rough surface and low resolution	Limited material, less biocompatible and limited open-source	Rough surface, less biocompatible and high costs

**Table 3:** Surface wettability of some printing materials used for fabrication of droplet microfluidic devices.

Printing Method	Material	Contact Angle
FDM	PLA	132 [103]
	ABS	81 [104]
	TPU	82 [105]
	Nylon 6-6	70 [106]
SLA	Formlabs clear resin	79 [107]
DLP	Kudo3D clear resin	78–85
Polyjet	Stratasys Veroclear resin	Glossy surface, 57
		Matte surface, 70

Provided by the company. Self-measured.
